# Socioeconomic determinants of psychotropic drug utilisation among elderly: a national population-based cross-sectional study

**DOI:** 10.1186/1471-2458-10-118

**Published:** 2010-03-09

**Authors:** Eva Lesén, Karolina Andersson, Max Petzold, Anders Carlsten

**Affiliations:** 1 Nordic School of Public Health (NHV), Box 121 33, 402 42 Gothenburg, Sweden; 2 Department of Research and Development, the National Corporation of Swedish Pharmacies, Gothenburg, Sweden

## Abstract

**Background:**

Psychotropic drugs are commonly utilised among the elderly. This study aimed to analyse whether two socioeconomic determinants - income and marital status - are associated with differences in utilisation of psychotropic drugs and potentially inappropriate psychotropic drugs among elderly in Sweden.

**Methods:**

All individuals aged 75 years and older who had purchased a psychotropic drug in Sweden during 2006 were included (68.7% women, n = 384712). Data was collected from national individual-based registers. Outcome measures were utilisation of three or more psychotropic drugs and utilisation of potentially inappropriate psychotropic drugs, as classified by the Swedish National Board of Health and Welfare.

**Results:**

Individuals with low income were more likely to utilise three or more psychotropic drugs compared to those with high income; adjusted odds ratio (aOR) 1.12 (95% confidence interval [CI] 1.10-1.14). The non-married had a higher probability for utilising three or more psychotropic drugs compared to the married (aOR 1.22; CI 1.20-1.25). The highest probability was observed among the divorced and the never married. Potentially inappropriate psychotropic drugs were more common among individuals with low compared to high income (aOR 1.14; CI 1.13-1.16). Compared to the married, potentially inappropriate psychotropic drug utilisation occurred more commonly among the non-married (aOR 1.08; CI 1.06-1.10). The never married and the divorced had the highest probability.

**Conclusions:**

There was an association between socioeconomic determinants and psychotropic drug utilisation. The probability for utilising potentially inappropriate psychotropics was higher among individuals with low income and among the non-married.

## Background

Psychotropic drugs are common among the elderly and represent a considerable proportion of inappropriate drugs used in this population [1]. The elderly are especially vulnerable to drug-related adverse health outcomes, and exposure to inappropriate drugs is associated with an increased risk of such events [2]. Socioeconomic determinants (e.g., poverty and lack of social support) influence health. Low socioeconomic status is associated with a higher prevalence of overall morbidity, including psychiatric morbidity [3-5], but also a decreased access to high-quality care and a lower consumer demand [6,7]. Further, marital status and social support is associated with health and health behaviour [8-10].

Health care quality differs between socioeconomic groups, particularly regarding the extent of preventive care, the cost of treatment procedures, and appropriate drug utilisation [6,11-13]. However, research focusing on the association between socioeconomic determinants, psychotropic drugs, and potentially inappropriate psychotropic drugs among the elderly is scarce [1,14]. Explicit criteria on potentially inappropriate drugs among the elderly often focus on pharmacological appropriateness, such as choice of drug, dose, drug interactions, duplications, and duration of drug therapy [15]. The Swedish National Board of Health and Welfare (NBHW) has published a list of quality indicators and explicit criteria for measuring inappropriate drug utilisation among the elderly in Sweden [16]. The list is based on Swedish recommendations, expert opinions, and internationally published explicit criteria such as Beers' criteria [17].

The introduction of the Swedish Prescribed Drug Register in 2005 [18] enabled linkage of information on drug utilisation and social determinants on an individual level with complete national coverage. This study aimed to analyse whether two socioeconomic determinants - income and marital status - are associated with differences in utilisation of psychotropic drugs and potentially inappropriate psychotropic drugs (PIP) among the elderly in Sweden.

## Methods

### Study participants and data sources

The study participants encompassed all individuals aged 75 years and older on 1 January 2006 in Sweden who had purchased a prescribed psychotropic drug in 2006 (n = 384712). Data on dispensed prescription drugs was obtained from the national individual-based Swedish Prescribed Drug Register held at the National Board of Health and Welfare [18]. The register includes all prescribed drugs purchased at Swedish pharmacies, but not drugs utilised in hospitals or purchased over the counter. Information on the participants' age, sex and all purchased prescription drugs (type, amount and date of purchase) was collected. In Sweden, co-payment for reimbursed drugs is independent of income. The maximum co-payment for prescription drugs within the pharmaceutical benefits scheme is SEK 1800 (SEK 1 = €0.106 on 1 January 2006) per twelve month period (Act 2002:160 on Pharmaceutical Benefits, etc.). Data from the Swedish Prescribed Drug Register was linked to the LISA database (the longitudinal integration database for health insurance and labour market studies; held at Statistics Sweden) via the unique person identification number. Information regarding family disposable income, number of family members, marital status, country of birth and date of death and migration was collected from the LISA database. The project was approved by the regional ethics board in Gothenburg, Sweden (No. 054-07).

Drugs were classified according to the Anatomical Therapeutic Chemical (ATC) classification system [19]. Psychotropic drugs were categorised as follows: any psychotropic (ATC-codes N05 and N06), antipsychotics (N05A), anxiolytics (N05B), hypnotics (N05C), and antidepressants (N06A).

### Socioeconomic determinants

Income was calculated using the square root scale (i.e., family disposable income during 2005 divided by the square root of the number of family members) [20,21]. The study participants were categorised into income tertiles to focus on any differences between those with the lowest income compared to those with the highest income. For analyses stratified by sex, this categorisation was performed separately for men and women. Marital status on 31 December 2006 was categorised as married, never married, divorced, or widowed. Country of birth was included as a potential confounder and categorised as Sweden, other Nordic countries, other European countries, and outside Europe. The number of unique nonpsychotropic drug substances purchased during the study period was used as a proxy for health care utilisation and comorbidity [22].

### Outcome measures

The number of unique psychotropic drugs purchased during 2006 was dichotomised into three or more vs. less than three psychotropic drugs. Utilisation of three or more psychotropic drugs was considered to reflect an increased exposure to psychotropic drugs. PIP was defined as the utilisation of potentially inappropriate psychotropic substances (PIPS) or potentially inappropriate combinations of psychotropics (PICP), as classified by the NBHW [16]. PIPS was defined as at least one purchase of long-acting benzodiazepines (flunitrazepam, nitrazepam, and diazepam), anticholinergic psychotropics, propiomazine, or triazolam during 2006. PICP was defined as concurrent utilisation of two or more benzodiazepines, two or more psychotropics in the same class (e.g., two or more hypnotics), and two or more anticholinergic psychotropics for a total of 40 days during 2006. The estimation of concurrent drug utilisation has been described previously [23]. Briefly, information on prescribers' dosage instructions is available only as a free text section in the register and is therefore not statistically processable. A review of prescribed daily doses (PDDs) was performed for a random sample of dispensed prescriptions for each substance. Substance-specific population average PDDs were estimated for each psychotropic substance, thus enabling the calculation of theoretical treatment periods based on date of purchase and purchased amount. A detailed description of PIP and associated prevalence rates have been reported previously [23].

### Statistical analyses

A person-year proportion, defined as the proportion of days resident in the country and alive during 2006, was calculated for each individual. For example, the person-year proportion for an individual who died on 26 March 2006 was 0.23 (84/365 days). The person-year proportion was included in the regression models, thereby taking date of death or migration into account. Further, the sum of the person-year proportions was used as the denominator when calculating the prevalence of utilisation of three or more psychotropic drugs and of PIP among the study participants.

Simple and multiple logistic regression analyses were performed for the outcome measures of three or more psychotropic drugs and PIP. The multiple logistic regression models included income or marital status, age, sex, country of birth, number of unique nonpsychotropic drug substances purchased during the study period, and the person-year proportion (not included in analyses on marital status). All regression analyses were also performed stratified by age and sex. Due to partially missing data, 366 individuals were excluded from the regression analyses including income. Marital status was recorded at the end of 2006 and was therefore missing for the 40880 individuals who died and the 77 who migrated during the study period. Further, the regression analyses were repeated in a 10% random sample, since the risk of false positive results increases as the sample size increases.

Simple and multiple Poisson regression analyses were performed for count outcomes (number of psychotropic drugs and number of PIP). The results from the Poisson regression analyses were in agreement with the logistic regression analyses, and are therefore not presented. Chi-square tests were used to compare proportions. The level for statistical significance was 0.05. SAS version 9.1.3 (SAS Institute, NC) was used for data management, and Stata version 10.1 (StataCorp, TX) was used for statistical analyses.

## Results

Table 1 includes the characteristics of the study participants. Individuals with low income had €12003 or less, and those with high income had €15171 or more. Among those who purchased psychotropic drugs, median income was €14937 for men and €12620 for women. In the entire Swedish population aged 75 years and older (n = 800129), median income was €17352 for men and €13546 for women (source: Statistics Sweden). The proportion of married individuals was lower among psychotropic drug users than in the corresponding entire Swedish population (men 60.8% [189616/311796], women 27.5% [133955/487305]; source: Statistics Sweden).

**Table 1:** Characteristics of the study participants.

	Men (n = 120426)	Women (n = 264286)	Total (n = 384712)	Death/migration, No. (Row %)
**Age**				
75-79 years, No. (%)	39636 (32.9)	74932 (28.4)	114568 (29.8)	6128 (5.4)
80-84 years, No. (%)	39728 (33.0)	80645 (30.5)	120373 (31.3)	9931 (8.3)
85+ years, No. (%)	41062 (34.1)	108709 (41.1)	149771 (38.9)	24898 (16.6)
*Missing, No. (%)*	0 (0.0)	0 (0.0)	0 (0.0)	0 (0.0)
Income (€)*				
High income, median (%)	21215 (33.0)	17457 (33.0)	18743 (33.0)	11106 (8.8)
Middle income, median (%)	14937 (33.8)	12620 (33.9)	13126 (33.9)	13438 (10.3)
Low income, median (%)	11748 (33.1)	10281 (33.0)	10632 (33.1)	16401 (12.9)
*Missing, No. (%)*	188 (0.2)	177 (0.1)	365 (0.1)	12 (3.3)
**Marital status**				
Married, No. (%)	58376 (48.5)	58395 (22.1)	116772 (30.4)	-
Never married, No. (%)	8400 (7.0)	13350 (5.1)	21750 (5.7)	-
Divorced, No. (%)	10100 (8.4)	25646 (9.7)	35746 (9.3)	-
Widowed, No. (%)	26814 (22.3)	142448 (53.9)	169262 (44.0)	-
*Missing, No. (%)†*	16736 (13.9)	24446 (9.2)	41182 (10.7)	40957 (99.5)
**Country of birth**				
Sweden, No. (%)	111738 (92.8)	241804 (91.5)	353542 (91.9)	38151 (10.8)
Other Nordic countries, No. (%)	3918 (3.3)	11852 (4.5)	15770 (4.1)	1429 (9.1)
Europe, No. (%)	3616 (3.0)	8232 (3.1)	11848 (3.1)	1067 (9.0)
Outside Europe, No. (%)	1130 (0.9)	2365 (0.9)	3495 (0.9)	310 (8.9)
*Missing, No. (%)*	24 (0.0)	33 (0.0)	57 (0.0)	0 (0.0)
**Number of unique drugs**				
1-4, No. (%)	12219 (10.1)	26479 (10.0)	38698 (10.1)	2489 (6.4)
5-9, No. (%)	41504 (34.5)	91856 (34.8)	133360 (34.7)	12040 (9.0)
10+, No. (%)	66703 (55.4)	145951 (55.2)	212654 (55.3)	26428 (12.4)
*Missing, No. (%)*	0 (0.0)	0 (0.0)	0 (0.0)	0 (0.0)

* Family disposable income for year 2005, adjusted for family size. SEK 1 = €0.106 (1 January 2006).

† Marital status was missing for all individuals who died or migrated during 2006 (n = 40957). When these were excluded, marital status was missing for 225 individuals.

Among the study participants aged 75-79 years, 22.5% (25828/114401) had low income; among individuals aged 85 years and older, the corresponding proportion was 43.7% (65422/149682). Death or migration occurred more commonly among individuals aged 85 years or older compared to 75-79 years (16.6% vs. 5.4%; p < 0.001), among those with low compared to high income (12.9% vs. 8.8%; p < 0.001), and among men compared to women (13.8% [16610] vs. 9.2% [24347]; p < 0.001) (Table 1).

Among elderly psychotropic drug users, 22.1% (85182) had purchased three or more psychotropic drugs during 2006. Individuals who died or migrated during the study period were more likely to utilise three or more psychotropic drugs compared to those who survived and did not migrate (31.8% [13019] vs. 21.0% [72163]; p < 0.001). Older age, female sex, and low income were associated with a higher probability for utilising three or more psychotropic substances (Table 2).

**Table 2:** Age, sex and income and utilisation of three or more psychotropic drugs among the study participants (n = 384346).

	Utilisation of three or more psychotropics, No./Total N (%)†	Odds Ratio (95% CI)‡	p-value	Adjusted Odds Ratio (95% CI)§	p-value
Age					
75-79	22729/111787 (20.3)	1 (Reference)		1 (Reference)	
80-84	26227/115802 (22.7)	1.12 (1.09-1.14)	< 0.001	1.09 (1.07-1.11)	< 0.001
85+	36226/137798 (26.3)	1.25 (1.22-1.27)	< 0.001	1.19 (1.16-1.21)	< 0.001
Sex					
Men	24671/112602 (21.9)	1 (Reference)		1 (Reference)	
Women	60511/252786 (23.9)	1.17 (1.15-1.19)	< 0.001	1.14 (1.12-1.16)	< 0.001
Income*					
High	25959/121784 (21.3)	1 (Reference)		1 (Reference)	
Middle	29087/124009 (23.5)	1.11 (1.09-1.13)	< 0.001	1.06 (1.04-1.08)	< 0.001
Low	30075/119233 (25.2)	1.19 (1.17-1.21)	< 0.001	1.12 (1.10-1.14)	< 0.001

* Family disposable income for year 2005, adjusted for family size.

† The denominator was adjusted according to the proportion of days the population was resident in the country and alive during 2006

‡ Adjusted for the proportion of days resident in the country and alive during 2006

§ Adjusted for age, sex, income, country of birth, proportion of days resident in the country and alive during 2006, and number of unique nonpsychotropic drug substances purchased during the study period.

Among individuals with low income, the adjusted odds ratio (aOR) for utilising three or more psychotropic drugs was 1.34 (95% confidence interval [CI] 1.26-1.43; reference: high income) for men aged 75-79 years, and 0.99 (CI 0.93-1.05) among men aged 85 years or more. For women, the corresponding aORs were 1.23 (CI 1.17-1.28) in the younger age group and 1.03 (CI 0.99-1.07) in the older age group.

Compared to married individuals, the never married, the divorced and the widows/widowers showed a higher probability for utilising three or more psychotropic drugs (Table 3). Men who were never married and divorced women showed the highest probability for utilising three or more psychotropic drugs. The associations were more pronounced among younger rather than older elderly (data not shown).

**Table 3:** Marital status and utilisation of three or more psychotropic drugs among the study participants (n = 343530)*.

	Utilisation of three or more psychotropics, No. (%)	Odds Ratio (95% CI)	p-value	Adjusted Odds Ratio (95% CI) †	p-value
**Total (n = 343530)**					
Married	21238 (18.2)	1 (Reference)		1 (Reference)	
Never married	5028 (23.1)	1.35 (1.31-1.40)	< 0.001	1.35 (1.30-1.40)	< 0.001
Divorced	8546 (23.9)	1.41 (1.37-1.45)	< 0.001	1.35 (1.31-1.39)	< 0.001
Widow/widower	37332 (22.1)	1.27 (1.25-1.30)	< 0.001	1.17 (1.14-1.19)	< 0.001
**Men (n = 103690)**					
Married	10119 (17.3)	1 (Reference)		1 (Reference)	
Never married	1897 (22.6)	1.39 (1.32-1.47)	< 0.001	1.45 (1.37-1.54)	< 0.001
Divorced	2165 (21.4)	1.30 (1.24-1.37)	< 0.001	1.32 (1.25-1.39)	< 0.001
Widow/widower	5489 (20.5)	1.23 (1.18-1.27)	< 0.001	1.19 (1.15-1.24)	< 0.001
**Women (n = 239840)**					
Married	11119 (19.0)	1 (Reference)		1 (Reference)	
Never married	3131 (23.5)	1.30 (1.25-1.36)	< 0.001	1.28 (1.23-1.34)	< 0.001
Divorced	6381 (24.9)	1.41 (1.34-1.46)	< 0.001	1.35 (1.30-1.40)	< 0.001
Widow/widower	31843 (22.4)	1.22 (1.19-1.25)	< 0.001	1.15 (1.12-1.18)	< 0.001

† Adjusted for age, sex, country of birth, and number of unique nonpsychotropic drug substances purchased during the study period

* Individuals who died or migrated during 2006 were excluded due to missing information on marital status

PIPS were utilised by 36.0% (138467), and PICP by 12.2% (46746); 9.0% (34596) utilised PIPS as well as PICP. Thus, PIP were utilised by 39.2% (150617). PIP utilisation occurred more commonly among those who died or migrated during the study period compared to those who survived and did not migrate (42.2% [17274] vs. 38.8% [133343]; p < 0.001, respectively). Older age and female sex were inversely associated with PIP utilisation, while low income was associated with a higher probability for PIP utilisation (Table 4). The probability for utilising PIPS and PICP, respectively, was higher among individuals with low income (PIPS: aOR 1.14; CI 1.12-1.16; PICP: aOR 1.11; CI 1.08-1.14; reference: high income). The aOR for having both PIPS and PICP was 1.12 (1.09-1.15) among individuals with low income compared to those with high income.

**Table 4:** Age, sex, and income and utilisation of potentially inappropriate psychotropics among the study participants (n = 384346).

	PIP, No./Total N (%)†	Odds Ratio (95% CI)‡	p-value	Adjusted Odds Ratio (95% CI)§	p-value
Age					
75-79	45788/111787 (41.0)	1 (Reference)		1 (Reference)	
80-84	46658/115802 (40.3)	0.95 (0.93-0.96)	< 0.001	0.93 (0.91-0.94)	< 0.001
85+	58171/137798 (42.2)	0.95 (0.93-0.96)	< 0.001	0.91 (0.90-0.93)	< 0.001
Sex					
Men	47997/112602 (42.6)	1 (Reference)		1 (Reference)	
Women	102620/252786 (40.6)	0.96 (0.95-0.97)	< 0.001	0.94 (0.93-0.95)	< 0.001
Income*					
High	48027/121784 (39.4)	1 (Reference)		1 (Reference)	
Middle	51491/124009 (41.5)	1.07 (1.06-1.09)	< 0.001	1.08 (1.07-1.10)	< 0.001
Low	50983/119233 (42.8)	1.10 (1.08-1.12)	< 0.001	1.14 (1.13-1.16)	< 0.001

PIP: Potentially inappropriate psychotropic drugs

* Family disposable income for year 2005, adjusted for family size.

† The denominator was adjusted for the proportion of days the population was resident in the country and alive during 2006

‡ Adjusted for the proportion of days resident in the country and alive during 2006

§Adjusted for age, sex, income, country of birth, proportion of days resident in the country and alive during 2006, and number of unique nonpsychotropic drug substances purchased during the study period.

The aOR among men with low income was 1.23 (95% CI 1.17-1.30; reference: high income) in the younger age group, and 1.14 (1.08-1.20) in the older age group. For women, the corresponding aORs were 1.13 (1.09-1.18) and 1.09 (1.05-1.12), respectively.

Never married and divorced individuals were more likely to utilise PIP compared to married individuals (Table 5). The probability for PIP utilisation was marginally higher among widows/widowers. These associations were more pronounced among the younger than the older elderly (data not shown). The aOR for having both PIPS and PICP was 1.51 (1.44-1.59) among the never married, 1.41 (1.36-1.47) among the divorced, and 1.14 (1.11-1.18) among the widows/widowers, as compared to the married.

**Table 5:** Marital status and utilisation of potentially inappropriate psychotropics among the study participants (n = 343530)*.

	PIP, No. (%)	Odds Ratio (95% CI)	p-value	Adjusted Odds Ratio (95% CI) †	p-value
**Total (n = 343530)**					
Married	44501 (38.1)	1 (Reference)		1 (Reference)	
Never married	9275 (42.6)	1.21 (1.17-1.24)	< 0.001	1.25 (1.21-1.29)	< 0.001
Divorced	15068 (42.2)	1.18 (1.16-1.21)	< 0.001	1.18 (1.15-1.21)	< 0.001
Widow/widower	64433 (38.1)	1.00 (0.98-1.01)	0.820	1.02 (1.01-1.04)	0.009
**Men (n = 103690)**					
Married	22303 (38.2)	1 (Reference)		1 (Reference)	
Never married	3755 (44.7)	1.31 (1.25-1.37)	< 0.001	1.35 (1.29-1.41)	< 0.001
Divorced	4378 (43.4)	1.24 (1.19-1.29)	< 0.001	1.24 (1.18-1.29)	< 0.001
Widow/widower	10420 (38.9)	1.03 (1.00-1.06)	0.068	1.04 (1.01-1.07)	< 0.001
**Women (n = 239840)**					
Married	22198 (38.0)	1 (Reference)		1 (Reference)	
Never married	5520 (41.4)	1.15 (1.11-1.19)	< 0.001	1.18 (1.14-1.23)	< 0.001
Divorced	10690 (41.7)	1.17 (1.13-1.20)	< 0.001	1.15 (1.11-1.18)	< 0.001
Widow/widower	54013 (37.9)	1.00 (0.98-1.02)	0.691	1.01 (0.99-1.03)	0.530

PIP: Potentially inappropriate psychotropic drugs

† Adjusted for age, sex, country of birth, and number of unique nonpsychotropic drug substances purchased during the study period

* Individuals who died or migrated during 2006 were excluded due to missing information on marital status

The regression analyses performed in the 10% random sample gave similar results as the original analyses, with two exceptions. The middle income group and the widows/widowers did not have a statistically significant higher probability for utilisation of PIP in the adjusted analyses (p = 0.08 and p = 0.66, respectively).

## Discussion

The present study showed a higher likelihood for utilising several psychotropics as well as PIP among individuals with low income and among the non-married. While the magnitudes of the higher probabilities were fairly small, they still indicate that socioeconomic determinants are associated with differences in utilisation of psychotropic drugs among the elderly. The magnitudes are comparable to a previous Swedish study on inappropriate drug use and education level among elderly [13]. These findings could suggest structural problems in the health care system.

Individuals with low income and the non-married were more likely to utilise three or more psychotropic drugs compared to those with high income and married individuals, respectively. Previous research has shown that the more vulnerable groups have an increased morbidity [4,5]. Information on morbidity was not available for this study; however, our findings could correspond to the ambition of the Swedish health care system to provide health care in relation to individual needs [24].

One of the cornerstones of the Swedish health care is that good health care should be provided for the entire population on equal terms [24]. Consequently, quality in drug treatment should not differ between socioeconomic groups. In this study, individuals with low income were more likely to utilise psychotropic drugs classified as potentially inappropriate by the NBHW compared to those with high income. These findings are consistent with previous studies on socioeconomic determinants and drug utilisation in Sweden [13] and in other countries [11,25-27]. Physicians' choice of drug may be influenced by patients' requests [28]. Since individuals in higher socioeconomic groups generally have increased access to health and drug information [6], they may be more likely to request drugs with a more favourable risk-benefit profile. Previous research has shown that individuals in higher socioeconomic groups are more likely to utilise newly marketed and brand-name drugs [26,29].

Marriage is positively associated with health and health behaviour [8-10]. In a somewhat younger population, divorced women and widowed men had an increased utilisation of psychotropic drugs compared to married or single men and women [14]. Married individuals in our study were less likely to utilise three or more psychotropic drugs as well as PIP compared to non-married individuals. Previous research on the utilisation of potentially inappropriate drugs and marital status or cohabitation is scarce and inconclusive [11,30]. The associations between marital status and drug utilisation in this population were more pronounced among men than among women, which may be related to the higher health benefits gained from marriage among men than among women [9,31].

Compared to women, men were less likely to utilise three or more psychotropic substances, but more likely to utilise PIP. Previous research showed that men were less likely to utilise antidepressants, but more likely to utilise a potentially inappropriate antidepressant [27]. Women generally are more knowledgeable than men about health issues, and physicians describe women as more demanding [32]. This characteristic could increase women's demands regarding quality of care. Further, the oldest old were more likely to utilise several psychotropic drugs, but less likely to utilise PIP as compared the younger elderly. The oldest old may have been prescribed these drugs for a long time and may be unwilling to change to newer drugs. Also, physicians may be more cautious in prescribing these drugs to the oldest old due to the increased risk of adverse events.

Individuals who died during the study period were more likely to utilise three or more psychotropics as well as PIP. This finding could indicate that these individuals had poorer health than the survivors, or that utilisation of these drugs might increase the risk for mortality. However, it is not possible to make any such conclusions based on the data available for this study.

The main strength of this study is its population-based design using national individual-based register data. However, our data does not include drugs utilised in hospitals. The most severe cases may therefore be omitted from the study population, although it is unlikely that individuals are hospitalised during periods long enough to avoid purchase of drugs in pharmacies, as the study period was one year. Further, no information on the actual use of drugs or indication was available. The cross-sectional design precludes any conclusions regarding causality. Marital status was recorded at the end of the study period; consequently, this information was missing for individuals who died or migrated during the study period. Those individuals were excluded from the regression analyses on marital status and PIP. Since marriage can be related to an increased survival [33], the association between marital status and PIP may be underestimated. Further, large sample sizes may increase the risk of false positive results. However, the regression analyses performed in the 10% random sample gave similar results as the original analyses.

Cost-related primary nonadherence is associated with income [34]. The Swedish Prescribed Drug Register includes purchased drugs only. Consequently, individuals who choose not to redeem their prescriptions, because they cannot afford them or for other reasons, are not present among the study participants. The restriction of the study participants to those with at least one purchase of a prescribed psychotropic drug may thus underestimate the relationship between income level and drug utilisation.

The classification of PIP was based on international explicit criteria such as Beers' criteria, adapted to Swedish conditions. The scientific documentation is substantial regarding the use of explicit criteria [2]. In some cases, however, drugs classified as potentially inappropriate may have been appropriately prescribed. This could not be determined, as the Swedish Prescribed Drug Register does not include the necessary information.

## Conclusions

The probability for utilising potentially inappropriate psychotropics, as classified by the NBHW, was higher among individuals with low income and among the non-married. These findings indicate that socioeconomic differences appear to exist in the quality of drug utilisation among elderly.

The differing levels of health knowledge and behaviour among the various socioeconomic groups need to be acknowledged in clinical practice. Efforts to increase good communication between prescribers and patients striving for concordance as well as patient empowerment should be promoted [35]. Further, drug prescribing should not be biased based on socioeconomic characteristics of the patient. Due to the increasing number of elderly in the population, an enhanced knowledge about interventions that effectively increases rational drug use and decreases the impact of socioeconomic determinants in the quality of care among the elderly is essential.

## Competing interests

The authors declare that they have no competing interests.

## Authors' contributions

All authors were responsible for study concept and design. EL and KA acquired the data. EL and MP were responsible for statistical analyses. All authors interpreted the data. EL drafted the manuscript and all authors contributed with critical revision of the manuscript. All authors read and approved the final manuscript.

## Pre-publication history

The pre-publication history for this paper can be accessed here:

http://www.biomedcentral.com/1471-2458/10/118/prepub
